# Altered parietal operculum cortex 2 functional connectivity in benign paroxysmal positional vertigo patients with residual dizziness: A resting‐state fMRI study

**DOI:** 10.1111/cns.14570

**Published:** 2024-02-07

**Authors:** Zhengwei Chen, Yueji Liu, Cunxin Lin, Dan Liu, Lijie Xiao, Haiyan Liu, Xiu‐e Wei, Liangqun Rong

**Affiliations:** ^1^ Department of Neurology Second Affiliated Hospital of Xuzhou Medical University Xuzhou China

**Keywords:** benign paroxysmal positional vertigo, functional connectivity, residual dizziness, resting‐state fMRI

## Abstract

**Aims:**

To investigate changes in functional connectivity (FC) focusing on parietal operculum cortex 2 (OP2) in benign paroxysmal positional vertigo (BPPV) patients with residual dizziness (RD) after successful canalith repositioning procedure (CRP).

**Methods:**

High‐resolution three‐dimensional T1 and resting‐state functional magnetic resonance imaging (fMRI) were performed on 55 healthy controls (HCs), 55 BPPV patients with RD, and 55 patients without RD after successful CRP. Seed‐based (bilateral OP2) FC was calculated to investigate the changes in FC among the three groups. Additionally, we further explored the associations between abnormal FC and clinical symptoms.

**Results:**

One‐way analysis of covariance showed significant FC differences among the three groups. Post‐hoc analysis showed that patients with RD exhibited decreased FC between left OP2 and regions of left angular gyrus (AG), thalamus, precuneus, middle frontal gyrus (MFG), and right cerebellum posterior lobe (CPL) in comparison with HCs. In addition, compared with patients without RD, patients with RD showed decreased FC between left OP2 and regions of left MFG, AG, middle temporal gyrus, and right CPL. Moreover, in patients with RD, the FC between left thalamus and OP2 was negatively correlated with duration of RD, and the FC between left AG and OP2 was negatively correlated with duration of BPPV.

**Conclusion:**

BPPV patients with RD showed reduced FC between brain regions involved in vestibular processing and spatial cognition; These results suggested that BPPV patients with RD might have diminished central processing of vestibular information and impaired spatial cognition.

## Graphical abstract

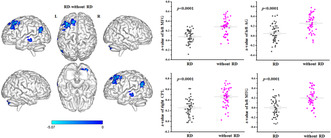

## 1 INTRODUCTION

Benign paroxysmal positional vertigo (BPPV) is the most common peripheral vertigo disease.^1^ Epidemiology showed that the lifetime prevalence of BPPV was 2.4%, the annual prevalence was 1.6%, and the annual incidence was 0.6%.^2^ Canalith repositioning procedure (CRP) is the most important treatment for BPPV, which removes the otolith from the affected semicircular canal and gets rid of vertigo instantly upon most occasions.^3^ However, 31% to 61% of BPPV patients still suffer from residual dizziness (RD) lasting a few weeks to several months after successful CRP. These BPPV patients with RD usually complain of a non‐specific dizziness, imbalance, or unsteadiness, without positional vertigo and nystagmus.^4^, ^5^

Currently, the potential neural mechanism of RD remains unclear. Some studies considered that the occurrence of RD was attributed to incomplete central nervous system adaptation and compensatory.^6^, ^7^ A recent resting‐state functional magnetic resonance imaging (fMRI) study suggested that patients with BPPV exhibited increased fractional amplitude of low‐frequency fluctuations (fALFF) in region of pons, functional changes in pons might reflect central adaptation and compensatory after repeated attacks of episodic vertigo.^8^ Previous studies have also shown that the vestibular nucleus of the pons might play an important role in central compensation in patients with unilateral external vestibular damage or in rats model of acute unilateral labyrinthotomy.^9^, ^10^ In central vestibular pathway, it was confirmed that the vestibular nucleus relies on vestibular information to the vestibular cortex through the thalamus.^11^ It was reported that the parieto‐insular vestibular cortex (PIVC) was the core of the human vestibular cortex, and parietal operculum cortex 2 (OP2) was the core of PIVC and was considered to be the primary vestibular cortical region in humans.^12^, ^13^ Thus, it is reasonable to think that the vestibular nucleus relies on vestibular information to the OP2 through the thalamus, and the functional changes in pons (vestibular nucleus) in patients with BPPV may indicate abnormal functional activity of the OP2. However, it is currently not clear whether BPPV patients with RD showed abnormal functional activity of the OP2.

Therefore, the present study aimed to evaluate the alterations in resting‐state functional connectivity (FC) focused on the bilateral OP2 and to determine whether these changes in FC correlate with certain clinical features in BPPV patients with RD after successful CRP. We hypothesize that the neural mechanism of RD involves functional changes in brain pathways through the OP2, and the altered FC of OP2 may potentially become an imaging biomarker for the diagnosis of RD in patients with BPPV.

## 2 MATERIALS AND METHODS

### 2.1 Participants

Between March 2020 and June 2023, 165 right‐handed subjects were recruited at the Second Affiliated Hospital of Xuzhou Medical University, including 55 BPPV patients with RD, 55 BPPV patients without RD, and 55 healthy volunteers. The BPPV patients were diagnosed using the criterion established by Bárány Society in 2015.^14^ Prior to enrollment, all patients underwent routine neuro‐otological interrogations and examinations to exclude secondary BPPV resulting from vestibular neuritis (VN), Meniere's disease (MD), vestibular migraine (VM), or head trauma. Patients with central nervous system disorders, psychiatric, or systemic disorders were excluded. BPPV with superior semicircular canal (SSC) or multi‐canal (MC) are rare. It was reported that SSC‐BPPV accounts for 1% to 3% of cases and the MC‐BPPV accounted for no more than 5% of patients with BPPV.^15^, ^16^ In addition, according to a recent systematic review and meta‐analysis, the affected side, location, or type of semicircular canal involvement have no concern with the occurrence of RD.^17^ Therefore, we only included BPPV patients with unilateral posterior semicircular canal (PSC) or horizontal semicircular canal (HSC); patients with MC or SSC were excluded.

Once diagnosed, patients with PSC‐BPPV were treated with Epley's maneuver^18^ or Semont maneuver^19^, ^20^ and patients with HSC‐BPPV were treated by Barbecue rotation maneuver (geotropic lateral canal BPPV)^21^ or Gufoni maneuver (apogeotropic lateral canal BPPV).^22^, ^23^ A Dix–Hallpike or supine‐roll test was performed 1 h after CRP to ensure a successful treatment.^24^, ^25^ After a successful CRP, the following demographic and clinical data were collected, including age, sex, educational years, affected side, involved semicircular canal, and duration of BPPV before successful CRP. In addition, scores of dizziness handicap inventory (DHI) and vertigo visual analog scale (VVAS) before successful CRM were collected. Then, all patients experienced 1 week follow‐up evaluation through interviews. During the interviews, the identification of RD symptoms was performed. Scores of DHI and dizziness VAS (DVAS) for patients with RD after successful CRP were recorded. All patients with and without RD underwent resting‐state fMRI scanning. In addition, the duration of RD symptoms for each patient with RD was collected. As symptoms of BPPV or RD may lead to anxiety and depression, some patients may even transition to persistent postural perceptual dizziness (PPPD, a chronic functional vestibular disorder which is characterized by persistent non‐rotational dizziness and instability combined with anxiety and depression lasting more than 3 months).^26^ All patients were assessed by Hamilton Anxiety Scale (HAMA) and Hamilton Depression Scale (HAMD) during the follow‐up; patients with scores of HAMA > 14 or HAMD > 17 were excluded. Patients with RD symptoms lasting more than 3 months were excluded.

Fifty‐five age‐, gender‐ and education‐matched volunteers were recruited. They had no history of vertigo, nor did they have a history of drug or alcohol abuse. Volunteers with neurological, mental, or systemic disorders were excluded. They received resting‐state fMRI scanning and demographic information was gathered. To rule out the effects of cognitive impairment on the brain function of the subjects, all subjects were evaluated by Montreal Cognitive Assessment (MoCA); participants with scores of MoCA < 26 were excluded. Assessments of MoCA, HAMA, and HAMD were conducted by a qualified neuropsychologist who was blinded to the outcome data. Our study was approved by the Ethics Committee of the Second Affiliated Hospital of Xuzhou Medical University (【2020】021801) and all subjects provided written informed consent forms before entering the study.

### 2.2 Imaging acquisition

All Participants received high‐resolution three‐dimensional T1‐weighted (3D‐T1) anatomical imaging and resting‐state fMRI using a 3.0T GE MRI scanner (GE Medical Systems) at the Second Affiliated Hospital of Xuzhou Medical University. Subjects were required to rest with their eyes closed and lie still during the scanning. The 3D‐T1 images were acquired with 1 mm isotropic voxels using a BRAVO sequence with the following parameters: repetition time (TR) = 2500 ms, echo time (TE) = 3.5 ms, flip angle (FA) = 8°, matrix = 256 × 256, 156 slices. We adopted a fast‐field echo‐planar imaging (EPI) sequence to acquire the whole brain fMRI images (TR = 2000 ms, TE = 30 ms, FA = 90°, field of view (FOV) = 200 × 200 mm, matrix = 64 × 64, thickness = 3.6 mm, gap = 0 mm, 210 volumes, total scan time = 7 min).

### 2.3 Image data preprocessing

To reduce the influence of initial unstable blood‐oxygenation‐level‐dependent (BOLD) signal, the first 10 functional time points were removed; the left 200 time points were preprocessed using Statistical Parametric Mapping 12 software (SPM12, http://www.fl.ion.ucl.ac.uk/spm/software/spm12) and CONN toolbox (Whitfeld‐Gabrieli S, 2012; Version 18b; http://www.nitrc.org/projects/conn) working on MATLAB R2016a (MathWorks, Inc.). The preprocessing steps were the default parameters within the CONN toolbox, including (a) functional slice‐timing correction, (b) functional realignment, (c) functional outlier detection (a subject head motion threshold set at 3 mm and a global signal threshold set at z = 9), (d) structural center to (0, 0, 0) coordinates (translation), (e) functional segmentation and normalization (DARTEL), and (f) spatial smoothing based on a Gaussian kernel of 6‐mm full‐width at half maximum. Four patients in RD group, five patients in non‐RD group, and two healthy subjects were excluded due to large head motion or poor normalization. Totally, 51 patients with RD, 50 patients without RD, and 53 healthy subjects were included in the following analysis.

### 2.4 Seed‐based functional connectivity

After preprocessing, filtering (0.01–0.08 Hz) was adopted to eliminate the influences of low‐frequency drift and high‐frequency noise. Cerebrospinal fluid, white matter, and global mean signals, as well as the 24 motion realignment parameters were regressed out. We performed a seed‐to‐voxel method using two seeds (the bilateral OP2) related to key vestibular cortex of the brain. We extracted the two seeds from the Juelich histological atlas available on the FMRIB Software Library (https://fsl.fmrib.ox.ac.uk/fsl/fslwiki/Atlases). Then, the mean time‐courses of the two seeds were extracted and the Pearson's correlation coefficients (*r*) between the extracted time‐courses and all other time‐courses of the entire brain voxels were calculated. Finally, *r* was converted to z scores using Fisher's r‐to‐z transformation. It is now still complex and controversial whether to remove the global mean signal. Therefore, we also performed an analysis without using global signal regression (GSR) and the results are demonstrated in Tables S1 and S2 and Figures S1–S3.

### 2.5 Statistical analysis

#### 2.5.1 Analysis of demography and clinical characteristics

Statistical analyses were performed using the Statistical Package for the Social Sciences (SPSS, v22.0) for Windows (SPSS Institute Inc.). All data of continuous variables were tested for normality. Group comparisons of demographic information among the three groups were performed using chi‐square tests for categorical variables (gender) and one‐way analysis of covariance (ANOVA) for parametric continuous variables (age, years of education, scores of MoCA, HAMA, and HAMD). For BPPV patients with and without RD, the two‐sample *t*‐tests were used for parametric continuous variables (scores of VVAS and DHI, and duration of vertigo before successful CRM) and chi‐square tests were used for categorical variables (affected side and affected semicircular canal).

#### 2.5.2 Analysis of differences in FC

Group differences in FC among the three groups were tested using a voxel‐wise one‐way ANOVA, with age, gender, years of education, scores of MoCA, HAMA, and HAMD as covariates. To show the inter‐group differences, we performed post‐hoc two‐sample *t*‐tests between every two of the three groups, controlling for demographic and clinical features severally. The post‐hoc inter‐group comparisons were performed within the mask which showed FC differences from the ANOVA analysis. Multiple comparisons were corrected by a false discovery rate (FDR) method (*p* < 0.05).

#### 2.5.3 Correlation analysis

For FC showing significant between‐group differences, we performed Pearson's partial correlation analysis between altered FC and clinical characteristics (duration of vertigo, scores of VVAS and DHI before successful CRM, and duration of RD, scores of DVAS and DHI after successful CRM) in BPPV patients with RD, controlling for age, gender, educational years, MoCA, HAMA, HAMD, affected side, and affected semicircular canal. The significance level was set at *p* < 0.05.

## 3 RESULTS

### 3.1 Demographic and clinical characteristics

The demographic and clinical characteristics of the three groups were summarized in Table 1. As we could see, there was no statistical difference among the three groups in age, gender, education level, scores of MoCA, HAMA, and HAMD (all *p >* 0.05). In addition, no significant difference was observed between BPPV patients with and without RD in affected side, affected semicircular canal, and VVAS scores before successful CRP (all *p* > 0.05). However, BPPV patients with RD showed longer duration of BPPV (*p* = 0.002) and higher DHI scores (*p* = 0.005) before successful CRP in comparison with BPPV patients without RD.

**TABLE 1:** Demographic and clinical characteristics of the subjects.

	RD (*n* = 51)	Without RD (*n* = 50)	HC (*n* = 53)	*p*‐value
Age (years)	54.17 ± 7.92	52.13 ± 7.16	50.38 ± 10.06	0.075
Gender (female/male)	40/11	35/15	31/22	0.088
Education (years)	10.54 ± 3.56	11.24 ± 3.39	11.40 ± 3.17	0.163
HAMA	9.40 ± 3.42	8.24 ± 3.16	7.36 ± 3.03	0.062
HAMD	8.79 ± 3.11	8.23 ± 2.35	7.17 ± 3.50	0.097
MoCA	26.94 ± 1.14	27.55 ± 1.29	28.12 ± 1.44	0.414
Affected canal (P/H)	38/13	43/7	NA	0.276
Affected side (L/R)	22/29	26/24	NA	0.373
Before successful CRP				
Duration of BPPV (days)	7.39 ± 2.71	3.44 ± 2.58	NA	0.002
Scores of DHI	48.58 ± 16.83	39.11 ± 11.97	NA	0.005
Scores of VVAS	6.74 ± 2.33	5.83 ± 2.12	NA	0.097
After successful CRP				
Duration of RD (days)	27.90 ± 17.42	0	NA	<0.0001
Scores of DHI	27.54 ± 11.46	0	NA	<0.0001
Scores of DVAS	3.28 ± 1.19	0	NA	<0.0001

Abbreviations: BPPV, Benign paroxysmal positional vertigo; CRP, Canalith repositioning procedure; DHI, Dizziness handicap inventory; DVAS, Dizziness visual analog scale; H, Horizontal; HAMA, Hamilton anxiety scale; HAMD, Hamilton depression scale; HC, Healthy control; L, Left; MoCA, Montreal cognitive assessment scale; NA, Not applicable; P, Posterior; R, Right; RD, Residual dizziness; VVAS, Vertigo visual analog scale.

### 3.2 FC differences across groups

**FIGURE 1:**
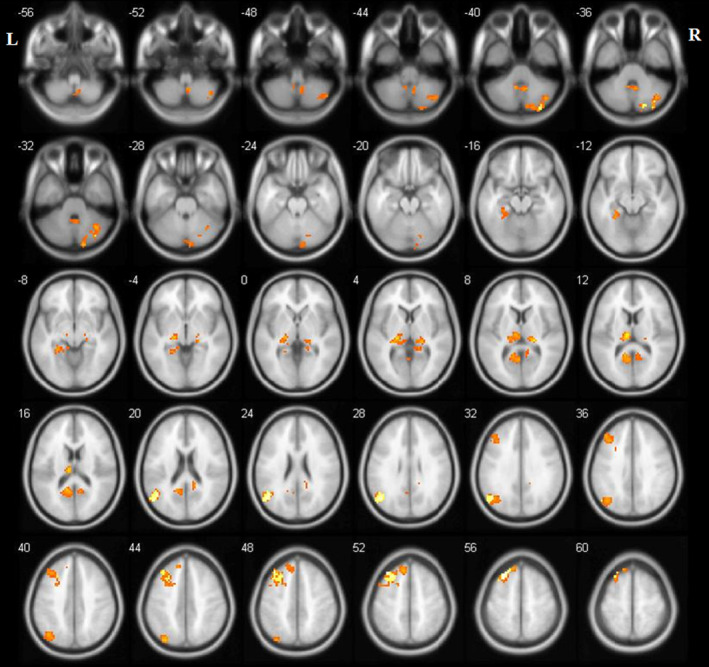
Brain regions showing significant FC differences among the three groups when the left OP2 was selected as a seed (one‐way ANOVA, *p* < 0.05 (FDR corrected)). ANOVA, Analysis of covariance; FC, Functional connectivity; FDR, False discovery rate; L, Left; OP2, parietal operculum cortex 2; R, Right.

**FIGURE 2:**
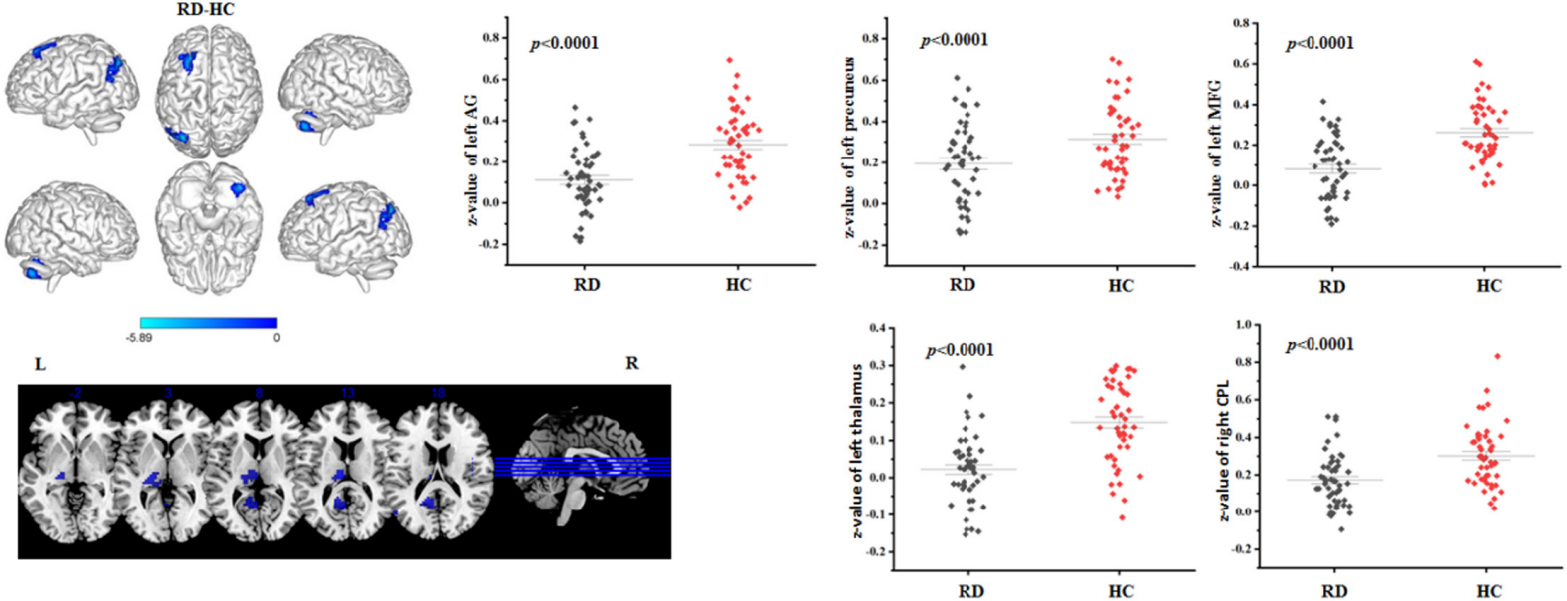
Differences in FC between BPPV patients with residual dizziness (RD) and healthy controls (HC) when the left OP2 was chosen as a seed (two‐sample *t* test, *p* < 0.05 (FDR corrected)). AG, Angular gyrus; BPPV, Benign paroxysmal positional vertigo; CPL, Cerebellum posterior lobe; FC, Functional connectivity; FDR, False discovery rate; L, Left; MFG, Middle frontal gyrus; OP2, parietal operculum cortex 2; R, Right.

**FIGURE 3:**
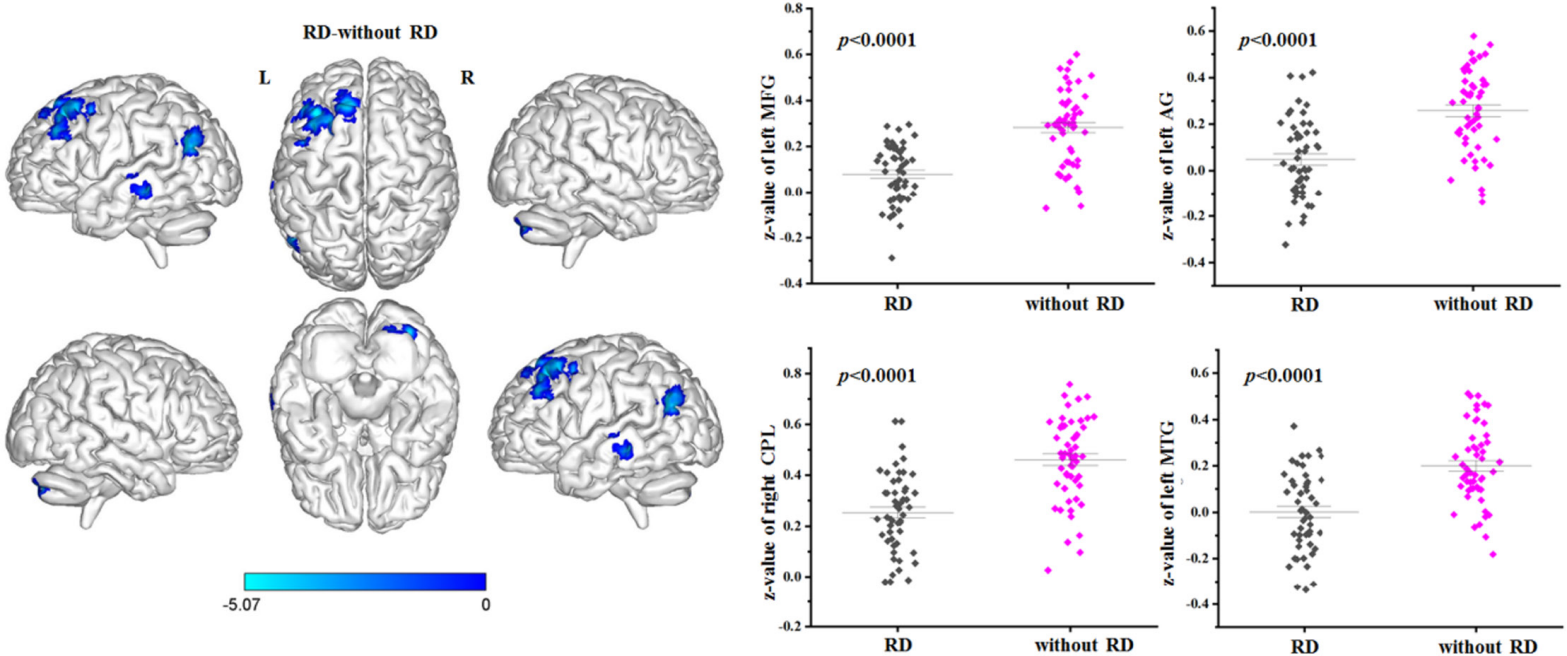
Differences in FC between BPPV patients with residual dizziness (RD) and patients without RD when the left OP2 was selected as a seed (two‐sample *t* test, *p* < 0.05 (FDR corrected)). AG, Angular gyrus; BPPV, Benign paroxysmal positional vertigo; CPL, Cerebellum posterior lobe; FC, Functional connectivity; FDR, False discovery rate; MFG, Middle frontal gyrus; MTG, Middle temporal gyrus; OP2, parietal operculum cortex 2.

When the left OP2 was selected as a seed, the three groups showed significant FC differences in the left angular gyrus (AG), left middle frontal gyrus (MFG), superior frontal gyrus (SFG), parahippocampa gyrus, and middle temporal gyrus (MTG), as well as the bilateral thalamus, precuneus, and cerebellar regions (*p* < 0.05, FDR corrected; Figure 1). When the right OP2 was chosen as a seed, one‐way ANOVA revealed no obvious difference among the three groups in FC. The post‐hoc results demonstrated that BPPV patients with RD exhibited decreased FC between the left OP2 and regions of the left AG, thalamus, precuneus, MFG, and the right cerebellum posterior lobe (CPL) in comparison with healthy subjects (*p* < 0.05, FDR corrected; Figure 2, Table 2); Beyond that, compared with BPPV patients without RD, patients with RD showed decreased FC between the left OP2 and regions of the left MFG, AG, MTG, and the right CPL (*p* < 0.05, FDR corrected; Figure 3, Table 3).

**TABLE 2:** Altered FC of the left OP2 in BPPV patients with RD compared with healthy controls.

Brain regions	Voxel size	Peak MNI coordinates *x*, *y*, *z*	Peak t‐score	AAL	BA
L AG	159	−42, −69, 34	−5.2017	Angular_L	39
L thalamus	137	−9, −18, 12	−5.8887	Thalamus_L	50
L precuneus	125	−12, −51, 18	−5.414	Precuneus_L	23
R CPL	102	36, –63, −30	−5.0222	Cerebelum_Crus2_R	37
L MFG	99	−24, 30, 57	−4.4711	Frontal_Mid_L	8

*Note*: The results were assigned thresholds at *p* < 0.05 (FDR corrected).

Abbreviations: AAL, Anatomical automatic labeling; AG, Angular gyrus; BA, Brodmann area; BPPV, Benign paroxysmal positional vertigo; CPL, Cerebellum posterior lobe; FC, Functional connectivity; FDR, False discovery rate; L, Left; MFG, Middle frontal gyrus; MNI, Montreal neurological institute; OP2, parietal operculum cortex 2; R, Right; RD, Residual dizziness.

**TABLE 3:** Abnormal FC of the left OP2 in patients with RD compared with patients without RD.

Brain regions	Voxel size	Peak MNI coordinates *x*, *y*, *z*	Peak t‐score	AAL	BA
L MFG	338	−36, 30, 51	−5.0699	Frontal_Mid_L	8
L AG	106	−45, −63, 24	−4.6648	Angular_L	39
R CPL	63	21, –84, −42	−4.4448	Cerebelum_Crus2_R	18
L MTG	51	−66, −27, −6	−4.8116	Temporal_Mid_L	21

*Note*: Significance was determined at *p* < 0.05 (FDR corrected).

Abbreviations: AAL, Anatomical automatic labeling; AG, Angular gyrus; BA, Brodmann area; CPL, Cerebellum posterior lobe; FC, Functional connectivity; FDR, False discovery rate; L, Left; MFG, Middle frontal gyrus; MNI, Montreal neurological institute; MTG, Middle temporal gyrus; OP2, parietal operculum cortex 2; R, Right; RD, Residual dizziness.

In results without GSR, the FC patterns of the OP2 with other brain regions were similar to the above results with GSR. The difference in results without GSR was that the left OP2 showed decreased FC with left fusiform gyrus in BPPV patients with RD compared with healthy controls, and the left OP2 displayed decreased FC with right superior parietal lobule and left inferior parietal lobule in BPPV patients with RD compared with patients without RD (*p* < 0.05, FDR corrected; Tables S1 and S2 and Figures S2 and S3).

### 3.3 Associations between FC and clinical characteristics in BPPV patients with RD

**FIGURE 4:**
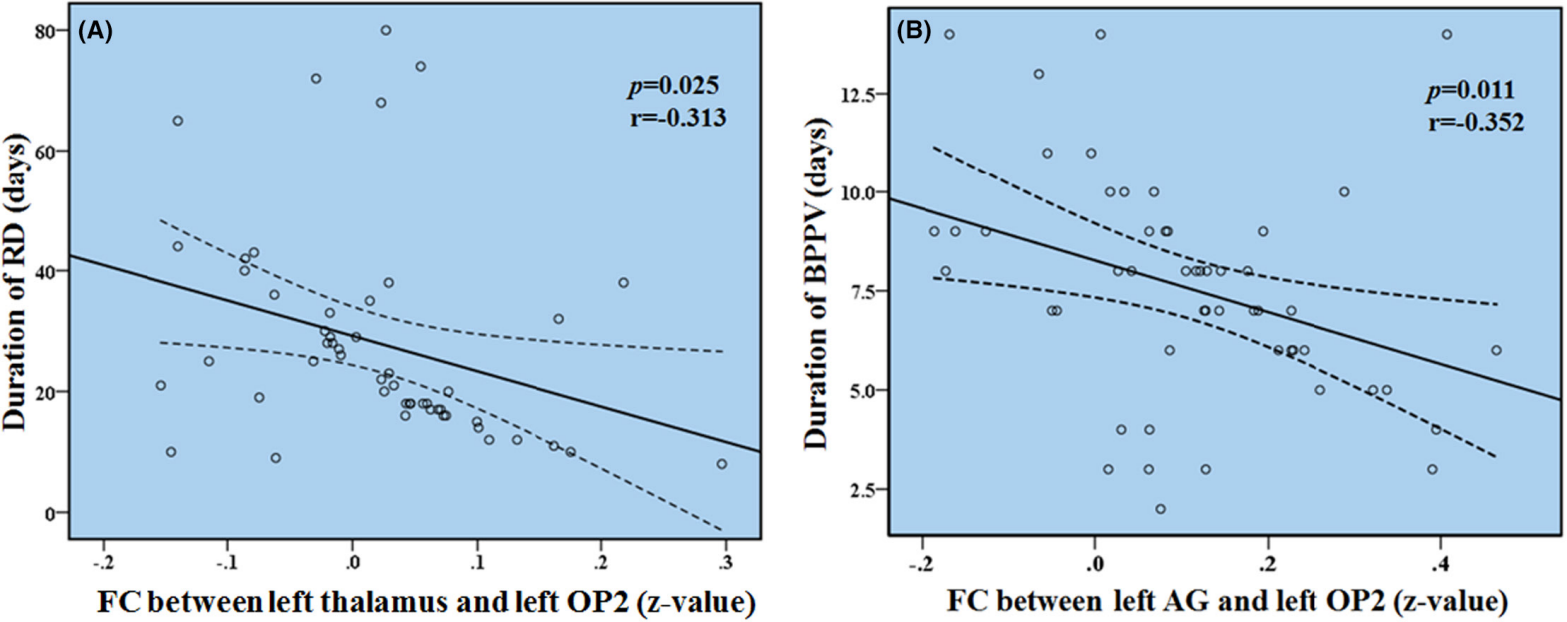
(A) Functional connectivity (FC) between the left thalamus and the left parietal operculum cortex 2 (OP2) was negatively correlated with the duration of residual dizziness (RD) (*p* = 0.025, *r* = −0.313); (B) FC between the left angular gyrus (AG) and the left OP2 was negatively correlated with the duration of benign paroxysmal positional vertigo (BPPV) in patients with RD (*p* = 0.011, *r* = −0.352).

As shown in Figure 4, in BPPV patients with RD, the FC (z‐value) between left thalamus and OP2 was negatively correlated with the duration of RD symptoms (*p* = 0.025, *r* = −0.313), and the FC (*z*‐value) between left AG and OP2 was negatively correlated with the duration of BPPV (*p* = 0.011, *r* = −0.352).

## 4 DISCUSSION

This study is the first one which investigated resting‐state FC changes focusing on OP2 in BPPV patients with RD after successful treatment. The results showed altered FC between the left OP2 and regions of the left AG, MFG, thalamus, precuneus, MTG, and the right CPL in BPPV patients with RD compared to BPPV patients without RD and healthy volunteers.

Compared with patients without RD and healthy subjects, patients with RD exhibited decreased FC between left OP2 and left AG. The AG is located in a posterior part of the inferior parietal lobule corresponding to Brodmann Area (BA) 39.^27^ A functional neuroimaging study suggested that the bilateral AG were significantly activated during task of spatial navigation.^28^ It was also reported that there existed structural plasticity in the AG when subjects were learning new skills that tap on spatial coordination.^29^ Thus, it is believed that the AG is involved in spatial cognition, which reflects our ability to process and integrate all spatial aspects of our environment, including the spatial analysis of external sensory information and internal mental representations.^30^ The functional changes in the left AG have been previously observed in patients with chronic unilateral vestibulopathy during rest and in patients with PPPD during stationary emotional stimulation.^31^, ^32^ Our study found altered function in AG in BPPV patients with RD, which was consistent with a previous resting‐state fMRI study which reported decreased ALFF in the AG in BPPV patients with RD compared to patients without RD.^33^ We also found that the FC between left AG and OP2 was negatively correlated with the duration of BPPV. Our results indicated impaired spatial cognition in BPPV patients with RD, and the occurrence of RD might be related to the reduced FC between the areas involved in central vestibular processing and spatial cognition.

We also found decreased FC between the left OP2 and right CPL/Crus2 in BPPV patients with RD compared to patients without RD and healthy controls. Central vestibular pathway involves the cerebellum, as the projections from the vestibular nuclei extend to the cerebellum.^34^ The cerebellum is an important area playing a vital role in maintaining balance and coordination of the goal‐oriented movements.^35^ The cerebellum is closely associated with dizziness and vertigo; stroke with lesions within the cerebellum is one of the most common causes of vascular vertigo.^36^ As part of the cerebellum, the CPL/Crus2 was reported to be closely related to spatial cognition, playing an important role in spatial navigation and orientation.^37^, ^38^ The altered function in CPL/Crus2 found in our study was in keeping with the abnormal gray matter volume (GMV) and regional homogeneity (ReHo) in CPL/Crus2 in patients with BPPV in a previous neuroimaging study.^8^ Thus, we suspected that the decreased FC between left OP2 and right CPL/Crus2 might also indicate reduced connectivity between the regions engaged in vestibular processing and spatial cognition.

The MFG was previously thought to be involved in vestibular processing and was reported to be a part of vestibular network, as it was obviously activated during tasks of vestibular or caloric stimulation.^39^, ^40^ The MFG receives vestibular information from vestibular nuclei and is believed to be the origin of direct white matter fibers to the vestibular nuclei.^41^ Functional changes in MFG have been reported in previous vestibular disorders, including VM, PPPD, visually induced dizziness (VID), and mal de debarquement syndrome.^42^, ^43^, ^44^, ^45^, ^46^ In BPPV patients with RD, we found decreased FC between left OP2 and left MFG when comparing to BPPV patients without RD and healthy controls. The decreased FC between OP2 and MFG in our study might suggest decreased connectivity within the central vestibular network.

Based on previous neuroimaging studies and lesion studies, the thalamus plays an important role in central vestibular processing as well, it receives vestibular input from the vestibular nuclei and the cerebellum, and conveys vestibular information to the central vestibular cortex.^39^, ^47^, ^48^, ^49^ In this study, we found decreased FC between left OP2 and left thalamus in BPPV patients with RD, indicating decreased thalamo‐OP2 vestibular pathway. In addition, we observed that the FC between left thalamus and OP2 was negatively correlated with duration of RD. As all fMRI data were collected at the early stage of RD symptoms (within 1 week), we believed that the decreased FC between left thalamus and OP2 potentially predict the development and the duration of residual dizziness in patients with BPPV.

The precuneus is a core region of default mode network (DMN) and plays a crucial role in attention monitoring and self‐centered cognition.^50^ It was demonstrated that electrical stimulation of the precuneus evoked symptom of vertigo, suggesting that the precuneus was an important brain region in processing vestibular information.^51^, ^52^ In the present study, we observed decreased FC between left OP2 and left precuneus in BPPV patients with RD compared to healthy controls; this result was in agreement with a previous resting‐state fMRI study which reported decreased ALFF in the bilateral precuneus in BPPV patients with RD.^33^ Our results of decreased FC between left OP2 and left precuneus in BPPV patients with RD might indicate decreased connectivity within the vestibular network.

### 4.1 Limitations

This study has certain limitations in some aspects. First, the sample size of the present study was relatively small and only resting‐state fMRI was adopted, a multi‐model MRI study with larger sample size should be considered in the following study. Second, we only adopted a seed‐based FC method, which could not overcome the defect of forcibly segmenting the brain atlas. Subsequent studies should combine seed‐based FC with other methods, for example, independent component analysis (ICA)‐based FC or functional network connectivity (FNC), dynamic FC or FNC, graph theory analysis, etc. Last but not the least, the results of seeds‐based FC with and without global signal regression was not entirely consistent. This might have some influence on the conclusion of this study.

## 5 CONCLUSIONS

In summary, the current study found that BPPV patients with RD showed reduced FC between brain regions involved in vestibular processing and spatial cognition. These results suggested that BPPV patients with RD might have diminished central processing of vestibular information and impaired spatial cognition. In addition, the reduced FC could potentially become an imaging biomarker for the early diagnosis of RD in patients with BPPV, which should be further verified by machine learning (ML) methods with more subjects in the future.

## AUTHOR CONTRIBUTIONS

Zhengwei Chen and Yueji Liu designed the study, analyzed the data, and wrote the main manuscript; Cunxin Lin, Dan Liu, and Lijie Xiao collected the data; Haiyan Liu and Xiu‐e Wei organized the data; Liangqun Rong revised the manuscript. All authors contributed to the article and approved the submitted version.

## FUNDING INFORMATION

This study was funded by Xuzhou Municipal Health Commission (No. XWKYHT20200010) and Health Commission of JiangSu province (H2023014).

## CONFLICT OF INTEREST STATEMENT

All the authors declare that they have no conflict of interest.

## Supporting information


Data S1:

## Data Availability

The data that support the findings will be available in China Clinical Trial Registry at https://www.chictr.org.cn/index.html following an embargo from the date of publication to allow for commercialization of research findings.

## References

[1] Kim HJ , Park J , Kim JS . Update on benign paroxysmal positional vertigo. J Neurol. 2021;268(5):1995‐2000. 33231724 10.1007/s00415-020-10314-7 PMC7684151

[2] von Brevern M , Radtke A , Lezius F , et al. Epidemiology of benign paroxysmal positional vertigo: a population based study. J Neurol Neurosurg Psychiatry. 2007;78(7):710‐715. 17135456 10.1136/jnnp.2006.100420 PMC2117684

[3] Bhattacharyya N , Gubbels SP , Schwartz SR , et al. Clinical practice guideline: benign paroxysmal positional vertigo (update). Otolaryngol Head Neck Surg. 2017;156(3_suppl):S1‐S47. 10.1177/0194599816689667 28248609

[4] Nuti D , Zee DS , Mandalà M . Benign paroxysmal positional vertigo: what we do and do not know. Semin Neurol. 2020;40(1):49‐58. 31935770 10.1055/s-0039-3402733

[5] Martellucci S , Stolfa A , Castellucci A , et al. Recovery of regular daily physical activities prevents residual dizziness after Canalith repositioning procedures. Int J Environ Res Public Health. 2022;19(1):490. 35010750 10.3390/ijerph19010490 PMC8744883

[6] Faralli M , Lapenna R , Giommetti G , Pellegrino C , Ricci G . Residual dizziness after the first BPPV episode: role of otolithic function and of a delayed diagnosis. Eur Arch Otorhinolaryngol. 2016;273(10):3157‐3165. 26926693 10.1007/s00405-016-3947-z

[7] Seo T , Shiraishi K , Kobayashi T , et al. Residual dizziness after successful treatment of idiopathic benign paroxysmal positional vertigo originates from persistent utricular dysfunction. Acta Otolaryngol. 2017;137(11):1149‐1152. 28681630 10.1080/00016489.2017.1347824

[8] Zhu Q , Chen W , Cui Y , et al. Structural and functional changes in the cerebellum and brainstem in patients with benign paroxysmal positional vertigo. Cerebellum. 2021;20(5):804‐809. 33547587 10.1007/s12311-021-01237-8

[9] Helmchen C , Klinkenstein JC , Krüger A , et al. Structural brain changes following peripheral vestibulo‐cochlear lesion may indicate multisensory compensation. J Neurol Neurosurg Psychiatry. 2011;82(3):309‐316. 20802221 10.1136/jnnp.2010.204925

[10] Zwergal A , Schlichtiger J , Xiong G , et al. Sequential [(18)F]FDG μPET whole‐brain imaging of central vestibular compensation: a model of deafferentation‐induced brain plasticity. Brain Struct Funct. 2016;221(1):159‐170. 25269833 10.1007/s00429-014-0899-1

[11] Kirsch V , Keeser D , Hergenroeder T , et al. Structural and functional connectivity mapping of the vestibular circuitry from human brainstem to cortex. Brain Struct Funct. 2016;221(3):1291‐1308. 25552315 10.1007/s00429-014-0971-x

[12] Indovina I , Bosco G , Riccelli R . Structural connectome and connectivity lateralization of the multimodal vestibular cortical network. Neuroimage. 2020;222:117247. 32798675 10.1016/j.neuroimage.2020.117247 PMC7779422

[13] Zu Eulenburg P , Caspers S , Roski C , et al. Metaanalytical definition and functional connectivity of the human vestibular cortex. Neuroimage. 2012;60(1):162‐169. 22209784 10.1016/j.neuroimage.2011.12.032

[14] von Brevern M , Bertholon P , Brandt T , et al. Benign paroxysmal positional vertigo: diagnostic criteria. J Vestib Res. 2015;25(3–4):105‐117. 26756126 10.3233/VES-150553

[15] Naples JG , Eisen MD . Surgical management for benign paroxysmal positional vertigo of the superior semicircular canal. Laryngoscope. 2015;125(8):1965‐1967. 25583673 10.1002/lary.25123

[16] Si L , Ling X , Li Z , Li K , Shen B , Yang X . Clinical characteristics of patients with multi‐canal benign paroxysmal positional vertigo. Braz J Otorhinolaryngol. 2022;88(1):89‐100. 32595078 10.1016/j.bjorl.2020.05.012 PMC9422682

[17] Ke YJ , Ma X , Jing YY , Diao T , Yu L . Risk factors for residual dizziness in patients with benign paroxysmal positional vertigo after successful repositioning: a systematic review and meta‐analysis. Eur Arch Otorhinolaryngol. 2022;279(7):3237‐3256. 35218384 10.1007/s00405-022-07288-9

[18] Hilton MP , Pinder DK . The Epley (canalith repositioning) manoeuvre for benign paroxysmal positional vertigo. Cochrane Database Syst Rev. 2014;8(12):CD003162. 10.1002/14651858.CD003162.pub3 PMC11214163 25485940

[19] Levrat E , van Melle G , Monnier P , Maire R . Efficacy of the Semont maneuver in benign paroxysmal positional vertigo. Arch Otolaryngol Head Neck Surg. 2003 Jun;129(6):629‐633. 12810466 10.1001/archotol.129.6.629

[20] Zhang X , Qian X , Lu L , et al. Effects of Semont maneuver on benign paroxysmal positional vertigo: a meta‐analysis. Acta Otolaryngol. 2017;137(1):63‐70. 27683970 10.1080/00016489.2016.1212265

[21] Escher A , Ruffieux C , Maire R . Efficacy of the barbecue manoeuvre in benign paroxysmal vertigo of the horizontal canal. Eur Arch Otorhinolaryngol. 2007;264(10):1239‐1241. 17520267 10.1007/s00405-007-0337-6

[22] Fu W , Han J , Chang N , et al. Immediate efficacy of Gufoni maneuver for horizontal canal benign paroxysmal positional vertigo (HC‐BPPV): a meta‐analysis. Auris Nasus Larynx. 2020;47(1):48‐54. 31151785 10.1016/j.anl.2019.05.002

[23] van den Broek EM , van der Zaag‐Loonen HJ , Bruintjes TD . Systematic review: efficacy of Gufoni maneuver for treatment of Lateral Canal benign paroxysmal positional vertigo with geotropic nystagmus. Otolaryngol Head Neck Surg. 2014;150(6):933‐938. 24627409 10.1177/0194599814525919

[24] Sumner A . The dix‐Hallpike test. J Physiother. 2012;58(2):131. 22613247 10.1016/S1836-9553(12)70097-8

[25] Lim HJ , Park K , Park HY , Choung YH . The significance of 180‐degree head rotation in supine roll test for horizontal canal benign paroxysmal positional vertigo. Otol Neurotol. 2013;34(4):736‐742. 23370558 10.1097/MAO.0b013e31827de2d1

[26] Staab JP , Eckhardt‐Henn A , Horii A , et al. Diagnostic criteria for persistent postural perceptual dizziness (PPPD): consensus document of the committee for the classification of vestibular disorders of the Bárány society. J Vestib Res. 2017;27(4):191‐208. 29036855 10.3233/VES-170622 PMC9249299

[27] Seghier ML . The angular gyrus: multiple functions and multiple subdivisions. Neuroscientist. 2013;19(1):43‐61. 22547530 10.1177/1073858412440596 PMC4107834

[28] Spreng RN , Mar RA , Kim AS . The common neural basis of autobiographical memory, prospection, navigation, theory of mind, and the default mode: a quantitative metaanalysis. J Cogn Neurosci. 2009;21(3):489‐510. 18510452 10.1162/jocn.2008.21029

[29] Jung RE , Segall JM , Jeremy Bockholt H , et al. Neuroanatomy of creativity. Hum Brain Mapp. 2010;31(3):398‐409. 19722171 10.1002/hbm.20874 PMC2826582

[30] Sack AT . Parietal cortex and spatial cognition. Behav Brain Res. 2009;202(2):153‐161. 19463696 10.1016/j.bbr.2009.03.012

[31] Si L , Cui B , Li Z , et al. Concurrent brain structural and functional alterations in patients with chronic unilateral vestibulopathy. Quant Imaging Med Surg. 2022;12(6):3115‐3125. 35655817 10.21037/qims-21-655 PMC9131349

[32] von Söhsten Lins EMD , Bittar RSM , Bazán PR , Amaro Júnior E , Staab JP . Cerebral responses to stationary emotional stimuli measured by fMRI in women with persistent postural‐perceptual dizziness. Int Arch Otorhinolaryngol. 2021;25(3):e355‐e364. 34377168 10.1055/s-0040-1716572 PMC8321645

[33] Fu W , Bai Y , He F , et al. The association between Precuneus function and residual dizziness in patients with benign paroxysmal positional vertigo. Front Neurol. 2022;13:828642. 35493847 10.3389/fneur.2022.828642 PMC9039311

[34] Roostaei T , Nazeri A , Sahraian MA , Minagar A . The human cerebellum: a review of physiologic neuroanatomy. Neurol Clin. 2014;32(4):859‐869. 25439284 10.1016/j.ncl.2014.07.013

[35] Delgado‐García JM . Structure and function of the cerebellum. Rev Neurol. 2001;33(7):635‐642. 11784952

[36] Kim HA , Yi HA , Lee H . Recent advances in cerebellar ischemic stroke syndromes causing vertigo and hearing loss. Cerebellum. 2016;15:781‐788. 26573627 10.1007/s12311-015-0745-x

[37] Iglói K , Doeller CF , Paradis AL , et al. Interaction between hippocampus and cerebellum crus I in sequence‐based but not place‐based navigation. Cereb Cortex. 2015;25:4146‐4154. 24947462 10.1093/cercor/bhu132 PMC4886832

[38] Stoodley CJ , Valera EM , Schmahmann JD . Functional topography of the cerebellum for motor and cognitive tasks: an fMRI study. Neuroimage. 2012;59(2):1560‐1570. 21907811 10.1016/j.neuroimage.2011.08.065 PMC3230671

[39] Stephan T , Deutschländer A , Nolte A , et al. Functional MRI of galvanic vestibular stimulation with alternating currents at different frequencies. Neuroimage. 2005;26:721‐732. 15955481 10.1016/j.neuroimage.2005.02.049

[40] Lobel E , Kleine JF , Bihan DL , Leroy‐Willig A , Berthoz A . Functional MRI of galvanic vestibular stimulation. J Neurophysiol. 1998;80(5):2699‐2709. 9819274 10.1152/jn.1998.80.5.2699

[41] Their P , Erickson RG . Vestibular input to visual‐tracking neurons in area MST of awaken rhesus monkeys. Ann N Y Acad Sci. 1992;656:960‐963. 1599231 10.1111/j.1749-6632.1992.tb25307.x

[42] Chen Z , Xiao L , Liu H , et al. Altered thalamo‐cortical functional connectivity in patients with vestibular migraine: a resting‐state fMRI study. Neuroradiology. 2022;64(1):119‐127. 34374821 10.1007/s00234-021-02777-w

[43] Zhe X , Zhang X , Chen L , et al. Altered gray matter volume and functional connectivity in patients with vestibular migraine. Front Neurosci. 2021;15:683802. 34305518 10.3389/fnins.2021.683802 PMC8297163

[44] Lee JO , Lee ES , Kim JS , et al. Altered brain function in persistent postural perceptual dizziness: a study on resting state functional connectivity. Hum Brain Mapp. 2018;39(8):3340‐3353. 29656497 10.1002/hbm.24080 PMC6866559

[45] Van Ombergen A , Heine L , Jillings S , et al. Altered functional brain connectivity in patients with visually induced dizziness. Neuroimage Clin. 2017;14:538‐545. 28331800 10.1016/j.nicl.2017.02.020 PMC5345975

[46] Cha YH , Chakrapani S , Craig A , Baloh RW . Metabolic and functional connectivity changes in mal de debarquement syndrome. PLoS ONE. 2012;7(11):e49560. 23209584 10.1371/journal.pone.0049560 PMC3510214

[47] Indovina I , Maffe V , Bosco G , et al. Representation of visual gravitational motion in the human vestibular cortex. Science. 2005;208:416‐419. 10.1126/science.1107961 15831760

[48] Dieterich M , Bartenstein P , Spiegel S , Bense S , Schwaiger M , Brandt T . Thalamic infarctions cause side‐specific suppression of vestibular cortex activations. Brain. 2005;128:2052‐2067. 15947061 10.1093/brain/awh551

[49] Dieterich M , Brandt T . Thalamic infarctions: differential effects on vestibular function in the roll plane (35 patients). Neurology. 1993;43:1732‐1740. 8414023 10.1212/wnl.43.9.1732

[50] Raichle ME . The brain's default mode network. Annu Rev Neurosci. 2015;38:433‐447. 25938726 10.1146/annurev-neuro-071013-014030

[51] Kahane P , Hoffmann D , Minotti L , Berthoz A . Reappraisal of the human vestibular cortex by cortical electrical stimulation study. Ann Neurol. 2003;54(5):615‐624. 14595651 10.1002/ana.10726

[52] Wiest G , Zimprich F , Prayer D , Czech T , Serles W , Baumgartner C . Vestibular processing in human paramedian precuneus as shown by electrical cortical stimulation. Neurology. 2004;62(3):473‐475. 14872035 10.1212/01.wnl.0000106948.17561.55

