# Supplementary material for: Altered parietal operculum cortex 2 functional connectivity in benign paroxysmal positional vertigo patients with residual dizziness: A resting‐state fMRI study
Source: CNS Neurosci Ther. 2024 Feb 7;30(2):e14570. doi: 10.1111/cns.14570 (PMC10850607; doi:10.1111/cns.14570)
Supplement: Supplementary file 1 — Data S1: [file CNS-30-e14570-s001.docx]

Supplementary Material

# Supplementary Tables

**Table S1** Altered FC of the left OP2 in BPPV patients with RD compared with healthy controls (without global signal regression)

| **Brain regions** | **Voxel size** | **Peak MNI coordinates**  **x, y, z** | **Peak t-score** | **AAL** | **BA** |
| --- | --- | --- | --- | --- | --- |
| L AG | 174 | -33 -78 45 | -5.3031 | Angular_L | 39 |
| L thalamus | 144 | -14 -23 6 | -5.9103 | Thalamus_L | 50 |
| L FG | 72 | -27 -42 -9 | -4.4458 | Fusiform_L | 37 |
| L precuneus | 136 | -10 -55 14 | -5.2462 | Precuneus_L | 23 |
| L MFG | 114 | -26 23 54 | -4.9541 | Frontal_Mid_L | 8 |
| R CPL | 108 | 38 -66 -40 | -4.9433 | Cerebelum_Crus2_R | 37 |
| R CPL | 88 | 9 -54 -39 | -4.3558 | Cerebelum_Crus9_R | 37 |

The results were assigned thresholds at *p*<0.05 (FDR corrected). FDR, False discovery rate; FC, Functional connectivity; OP2, parietal operculum cortex 2; BPPV, Benign paroxysmal positional vertigo; RD, Residual dizziness; MNI, Montreal neurological institute; AAL, Anatomical automatic labeling; BA, Brodmann area; L, Left; R, Right; AG, Angular gyrus; FG, fusiform gyrus; CPL, Cerebellum posterior lobe; MFG, Middle frontal gyrus.

**Table S2** Abnormal FC of the left OP2 in patients with RD compared with patients without RD (without global signal regression)

| **Brain regions** | **Voxel size** | **Peak MNI coordinates**  **x, y, z** | **Peak t-score** | **AAL** | **BA** |
| --- | --- | --- | --- | --- | --- |
| L MFG | 558 | -30 25 44 | -5.3707 | Frontal_Mid_L | 8 |
| L AG | 159 | -49 -63 26 | -4.8350 | Angular_L | 39 |
| R CPL | 145 | 20 -82 -36 | -4.6741 | Cerebelum_Crus2_R | 18 |
| R SPL | 116 | 24 -54 48 | -4.4243 | Parietal_Sup_R | 7 |
| L IPL | 102 | -36 -54 54 | -4.5480 | Parietal_Inf_L | 7 |
| L MTG | 86 | -62 -30 -5 | -4.3535 | Temporal_Mid_L | 21 |

Significance was determined at *p*<0.05 (FDR corrected); FDR, False discovery rate; FC, Functional connectivity; OP2, parietal operculum cortex 2; RD, Residual dizziness; MNI, Montreal neurological institute; AAL, Anatomical automatic labeling; BA, Brodmann area; L, Left; R, Right; MFG, Middle frontal gyrus; AG, Angular gyrus; CPL, Cerebellum posterior lobe; SPL, Superior parietal lobule; IPL, Inferior parietal lobule; MTG, Middle temporal gyrus.

# Supplementary Figures


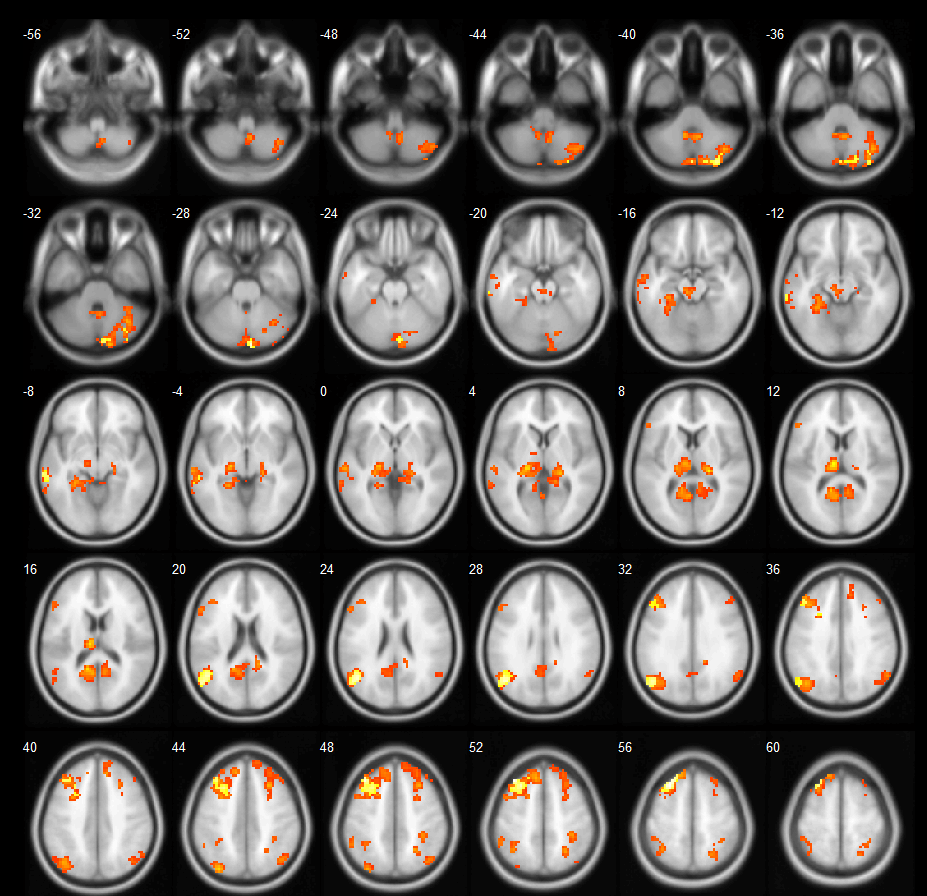


**Figure.S1** Brain regions showing significant FC differences among the three groups when the left OP2 was selected as a seed (without global signal regression, one-way ANOVA, *p*<0.05 (FDR corrected)). L, Left; R, Right; FC, Functional connectivity; OP2, parietal operculum cortex 2; ANOVA, Analysis of covariance; FDR, False discovery rate.


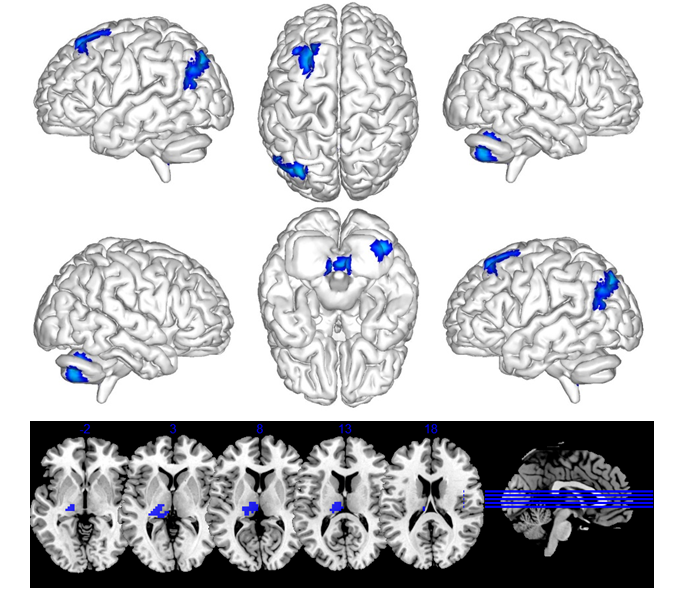
 **Figure.S2** Differences in FC between BPPV patients with residual dizziness (RD) and healthy controls (HC) when the left OP2 was chosen as a seed (without global signal regression, two-sample t test, *p*<0.05 (FDR corrected)). FC, Functional connectivity; BPPV, Benign paroxysmal positional vertigo; OP2, parietal operculum cortex 2; FDR, False discovery rate.


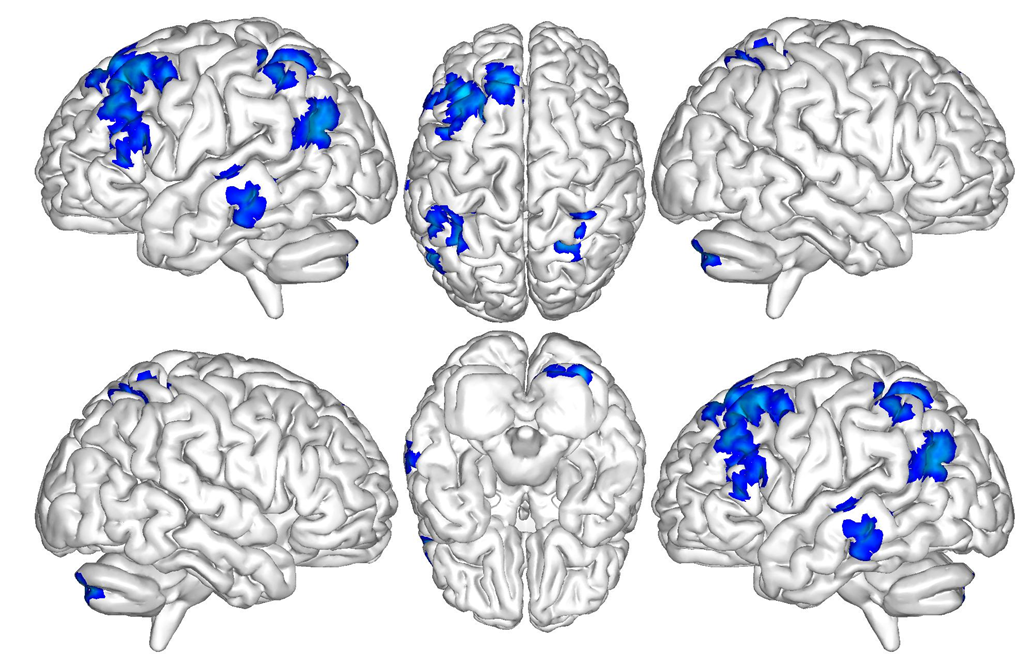


**Figure.S3** Differences in FC between BPPV patients with residual dizziness (RD) and patients without RD when the left OP2 was selected as a seed (without global signal regression, two-sample t test, *p*<0.05 (FDR corrected)). FC, Functional connectivity; BPPV, Benign paroxysmal positional vertigo; OP2, parietal operculum cortex 2; FDR, False discovery rate.
